# Nanostructured N, S, and P-Doped Elaeagnus Angustifolia Gum-Derived Porous Carbon with Electrodeposited Silver for Enhanced Electrochemical Sensing of Acetaminophen

**DOI:** 10.3390/nano13091571

**Published:** 2023-05-08

**Authors:** Xamxikamar Mamat, Haji Akber Aisa, Longyi Chen

**Affiliations:** Key Laboratory of Chemistry of Plant Resources in Arid Regions, State Key Laboratory Basis of Xinjiang Indigenous Medicinal Plants Resource Utilization, Xinjiang Technical Institute of Physics and Chemistry, Chinese Academy of Sciences, Urumqi 830011, China

**Keywords:** acetaminophen, nanostructured porous carbon, electrodeposition, silver enhancement, modified GCE, electrochemical sensing

## Abstract

Acetaminophen (N-acetyl-p-aminophenol, APAP) is regularly used for antipyretic and analgesic purposes. Overdose or long-term exposure to APAP could lead to liver damage and hepatotoxicity. In this study, the approach of enhanced electrochemical detection of APAP by nanostructured biomass carbon/silver was developed. Porous biomass carbon derived from Elaeagnus Angustifolia gum was prepared by pyrolysis with co-doping of electron-rich elements of nitrogen, sulfur, and phosphorus. The electrodeposition of silver onto a glassy carbon electrode modified with porous carbon could enhance the sensing signal towards APAP. Two linear ranges from 61 nM to 500 μM were achieved with a limit of detection of 33 nM. The developed GCE sensor has good anti-interference, stability, reproducibility, and human urine sample analysis performance. The silver-enhanced biomass carbon GCE sensor extends the application of biomass carbon, and its facile preparation approach could be used in constructing disposable sensing chips in the future.

## 1. Introduction

Acetaminophen (N-acetyl-p-aminophenol, APAP) is widely used for antipyretic and analgesic purposes, and careless handling of APAP could cause pollution in the environment. Overdose or exposure to APAP (4 g) could cause liver damage [1,2]. Additionally, APAP might have other unknown harmful effects on humans, animals, plants, etc. [3] For example, a study [4] by Melissa M. Smarr from the National Institutes of Health (NIH, Bethesda, MD, USA) found that a higher concentration of APAP in male urine would result in a longer time for pregnancy. While the concentration of APAP in female urine did not show a correlation with pregnancy. Rapid determination of APAP is very important in assisting its proper control and management for clinical diagnosis or environmental protection [5]. There are multiple methods for sensing APAP. For example, high-performance liquid chromatography, titrimetric, spectrophotometric, capillary electrophoresis, luminescence, and electrochemical methods [6]. In comparison with other analytical techniques, electrochemical methods have the advantages of quick response, high sensitivity, and good selectivity. APAP has redox peaks between about 0.3 V and 0.5 V, as the APAP molecule could be reduced and oxidized in this potential range.

With good electrochemical properties, biomass carbon has attracted attention recently and has been widely used for electrochemical sensing [7]. Biomass carbon could be prepared from plant or animal components, like leaves, fruit peels, pulp, animal feathers, etc. The major components of biomass are carbon and oxygen. Based on raw materials, some biomass carbon may contain metal elements. A high specific surface area is another characteristic of biomass carbon [8]. Therefore, biomass carbon often has good electrical conductivity and excellent electrochemical catalytic properties. Further modification of biomass carbon is also facile to perform, like doping elements and hybridizing nanomaterials, etc. The Zhou group [9] used gourd and milk as biomass sources to prepare worm-like nitrogen-doped carbon electrode material for electrochemical sensing of toxic heavy metal lead (II) ions. This modified GCE sensor exhibits a wide linear range from 0.5 μg L^−1^ to 100 μg L^−1^ and a low detection limit of 0.2 μg L^−1^. The Song group [10] prepared a three-dimensional macroporous carbon derived from kenaf stem as a sensing platform incorporated with Prussian blue and Cu and Co nanoparticles. This hybridized nanomaterial electrode material was used for sensing hydrogen peroxide, glucose, N-acetyl cysteine, and cysteine, which proves the potential application of this porous biomass carbon as a supporting platform for electrochemical sensing. The Chen group [11] utilized teak (Tectona grandis) leaves to prepare biomass carbon material integrated with Cu-Pd alloy nanoparticles for electrochemical sensing of dimetridazole. The GCE sensor modified by the biomass carbon/Cu-Pd alloy nanoparticles obtained a linear range of 0.15–119.4 μM and 144.4–746.9 μM, a limit of detection of 0.015 μM, and an analytical sensitivity of 0.79 μA μM^−1^ cm^−2^. The Rao group [12] fabricated CoFe-CoFe_2_O_4_ particles embedded in loofah sponges derived from biomass carbon with a molecular imprinted polymer film decorated glassy carbon electrode (GCE) sensor, which has a good linear relationship with the thiamphenicol concentrations in the range of 0.01–5000 μM with an ultralow detection limit of 0.003 μM.

The combination of noble nanomaterials into electrode materials could enhance the electrochemical signal due to their good conductivity and facile synthesis protocol [13]. Silver nanomaterials could be prepared from AgNO_3_ easily by reducing NaBH_4_ with a reductant, and silver-nanomaterial-modified electrodes for electroanalysis have been extensively studied [14]. The Xu group [15] combined the hybridization-inducing aggregate of DNA-functionalized silver nanoparticles with differential pulse stripping voltammetry detection for sensing platelet-derived growth factor. The silver nanoparticle aggregate stripping measurement offered signal amplification with a linear relationship in the range from 5 pg mL^−1^ to 1000 ng mL^−1^, and a limit of detection of 1.6 pg mL^−1^. The Shim group [16] synthesized a hydrazine-Au nanoparticle-aptamer bioconjugate and utilized the deposited silver stripping signal for electrochemical sensing of human epidermal growth factor receptor 2 (HER2) and the SK-BR-3 breast cancer cells overexpressing HER2. A good linearity was achieved in a dynamic range between 0.1 pg mL^−1^ and 10 ng mL^−1^ for HER2. The HER2 detection limit was determined to be 0.037 ± 0.002 pg mL^−1^. For sensing SK-BR-3 breast cancer cells, the linear range is between 50 and 20,000 cells mL^−1^, with a limit of detection of 26 cells mL^−1^. The Lu group [17] prepared several metal organic frameworks as electrode materials deposited with silver for hydrogen peroxide electrochemical sensing. The GCE sensor showed two wide linear ranges of 5 μM to 7 mM and 7 mM to 67 mM, with a low detection limit of 1.1 μM.

Recent studies of sensing APAP focused on using various nanomaterials and nanostructures that have high catalytic properties towards APAP. The Jiang group [18] engineered a hollow nanoporous carbon polyhedron embedded with Co/Co_3_O_4_ nanoparticles from the pyrolysis of a zeolitic imidazolate framework-67 containing Co elements. This modified GCE could utilize the catalytic property of Co/Co_3_O_4_ nanoparticles towards APAP and obtain two linear ranges of 0.025–2.5 μM and 2.5–50 μM with a low detection limit of 0.0083 μM. The Kumar group [19] fabricated GCE modified with N and P-doped hollow mesoporous carbon spheres, which displayed a wide linear sensing range from 5 μM to 1200 μM for APAP with a limit of detection of 0.02 μM. The Banks group [20] synthesized bismuth oxide nanorods and cast them onto screen-printed electrodes as a disposable sensor, which displayed strong electrocatalytic activity towards sensing APAP. A linear range from 0.5 μM to 1250 μM was achieved, along with a limit of detection of 30 nM.

Herein, we prepare a porous biomass carbon material derived from Elaeagnus Angustifolia gum. The major chemical composition of the gum is heteropolysaccharide. In order to enhance the biomass carbon’s electrical property, electron-rich elements of N, S, and P were doped into the material, and after a high-temperature pyrolysis procedure, the biomass carbon formed a porous nanostructure with a large specific surface area, offering plenty of active sites for catalyzing APAP molecules. Our group previously prepared Elaeagnus Angustifolia gum-derived porous carbon for sensing aflatoxin B_1_ [21], metronidazole [22], and chloramphenicol [23]. Good sensing performance was achieved for targeted analytes, which demonstrated the potential application of Elaeagnus Angustifolia gum-derived porous carbon in the electrochemical area. The facile preparation and modification approaches guarantee their potential usage as a nanostructured carbon electrode material. Considering a further enhancement of the biomass carbon’s sensing capability, silver was selected for in situ electrodeposition onto the biomass carbon surface. Therefore, a facilely prepared nanostructured Ag/biomass carbon@GCE electrochemical sensor was engineered, and a good sensing performance of APAP was achieved. The electrochemical sensing principle is the electrocatalytic oxidation of APAP into N-acetyl-4-benzoquinone imine along with the loss of two hydrogen ions and two electrons. This reaction is reversible by the cyclic voltammetry (CV) scan, a pair of redox peaks could be found in the CV curves.

## 2. Materials and Methods

Reagents and Apparatus. All chemicals were of analytical grade and used as received. Acetaminophen and Phosphate Buffered Saline (PBS) tablets are from Aladdin. The PBS solution used throughout the experiments is prepared using the PBS tablet. Sodium hypophosphite, thiocyanuric acid, and uric acid are from Macklin. Manganese sulfate is from Solarbio. Urea is from the Tianjin Tianda Fine Chemical Plant. Silver nitrate is from the Shanghai Fine Chemical Materials Research Institute. Potassium chloride is from Tianjin Bodi Chemical Co., Ltd. Ascorbic acid and potassium ferricyanide (III) are from the Tianjin Baishi Chemical Co., Ltd. Glucose and potassium hexacyanoferrate (II) trihydrate are from the Tianjin Hedong District Hongyan Reagent Factory. All solutions were prepared with water (18.2 MΩ cm) from a Millipore system. Dried Elaeagnus Angustifolia gum was provided by the Key Laboratory of Chemistry of Plant Resources in Arid Regions, State Key Laboratory Basis of Xinjiang Indigenous Medicinal Plants Resource Utilization. A human urine sample was provided by a healthy male volunteer in the lab. The volunteer did not consume any medicine containing APAP for at least a month before providing urine. A CHI-760E electrochemical workstation (Shanghai, Chenhua, China) and a RST 5000C workstation (Zhengzhou Shiruisi Technology Co., Ltd., Zhengzhou, China) were used in the electrochemical experiments. A field-emission scanning electron microscope (SEM, JEOL JSM-7610F Plus) equipped with an energy-dispersive X-ray (EDX) spectrometer was used to characterize the morphology and elemental composition of the electrode material.

Preparation of N-, S-, and P-doped porous carbon (NSP–PC). The NSP–PC was prepared by the method with a slight modification according to reference [21]. Elaeagnus gum (0.35 g) and manganese sulfate (0.45 g) were added to 5 mL of deionized water and mixed well as solution A. Then, urea (0.55 g), sodium hypophosphite (0.45 g), and thiocyanuric acid (0.35 g) were dispersed in another 5 mL of deionized water as solution B. Solution A and solution B were mixed together and set in the oven at 130 °C for 6 h to obtain a dried yellow powder. The yellow powder was carbonized at 800 °C for 3 h with a heating rate of 5 °C min^−1^ under N_2_ flow. The carbonized product was washed with a 15 wt. % HCl solution to remove metallic oxide and metal ion residues. The sample was centrifuged, washed several times with deionized water, and dried in a vacuum at 60 °C overnight to obtain the NSP–PC.

Fabrication of the Electrodes. Before modification, the GCE (3 mm in diameter) was polished with 0.5 μm and 0.05 μm alumina slurries to obtain a mirror-like surface. The electrode was then cleaned in ethanol and water by sonication and dried in an ambient environment. NSP–PC aqueous solution (2 mg mL^−1^) was sonicated first, and then 5 μL of the solution was dropped onto the GCE surface and dried in an ambient environment. The electrodeposition of silver was prepared in the aqueous AgNO_3_ (1 mg mL^−1^) using the three-electrode system under a 25 mV s^−1^ scan rate from 0 to −1 V of 3 CV cycles. After electrodeposition, the Ag/NSP–PC@GCE was rinsed with water and dried in an ambient environment.

Electrochemical measurements. The three-electrode system was used for the CV, DPV (differential pulse voltammetry), and electrodeposition, with SCE (saturated calomel electrode), platinum wire, and GCE. For EIS (electrochemical impedance spectroscopy) measurement, a three-electrode system of SCE (saturated calomel electrode), platinum plate electrode (10 mm × 10 mm × 0.1 mm), and GCE was used.

## 3. Results and Discussion

### 3.1. Material Characterization

#### Scanning Electron Microscopy and Energy Dispersive X-ray Characterization

**Figure 1:**
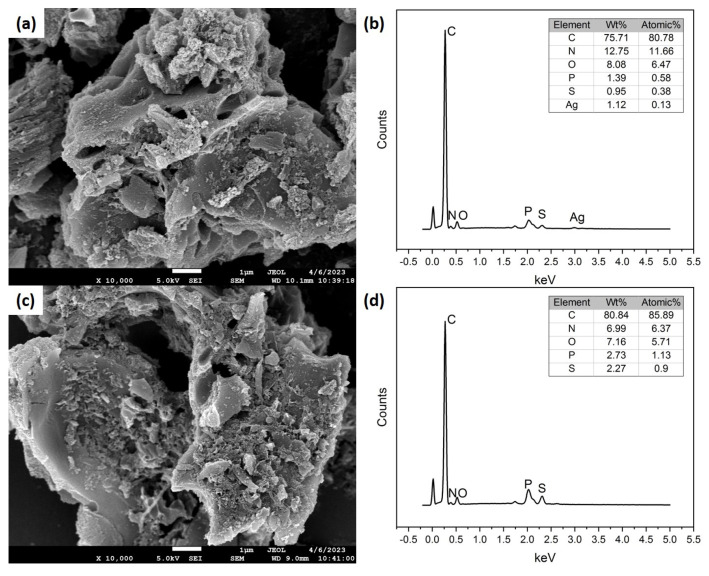
(**a**) is the Ag/NSP–PC@GCE, (**b**) is the corresponding EDX spectrum of (**a**), (**c**) is the NSP–PC@GCE, and (**d**) is the corresponding EDX spectrum of (**c**); the scale bar in (**a**,**c**) is 1 μm.

A custom-made GCE with a removable tip was adopted for the SEM and EDX characterizations. From the SEM images in Figure 1, the NSP–PC displayed a rough surface and porous structure, which were formed by the pore-forming chemical MnSO_4_ assisted by the pyrolysis procedure. The pore size varied from nanometers to micrometers, which resulted in a large specific surface area and offered lots of active spots for the catalytic reaction of APAP. The EDX spectra in Figure 1 confirmed the successful deposition of silver from aqueous AgNO_3_ by negative potential CV scans. The EDX spectrum of Ag/NSP–PC@GCE showed elements of C, N, O, P, S, and Ag, while the EDX spectrum of NSP–PC@GCE lacked the Ag signal. Because in the silver electrodeposition process, three cycles of CV scanning were performed, the Ag ratio was not high, with a weight ratio of 1.12% and an atomic ratio of 0.13%. Special silver nanostructures could be formed by controlling the electrodeposition conditions. The Pathan group [24] fabricated silver dendritic nanostructures using AgNO_3_ and a high concentration of citric acid. A potential of 1.5 V was used with changing deposition times of 100 s to 1200 s to form different silver nanostructures. With a shorter deposition time, a less dense silver dendritic nanostructure was formed. The 1200 s deposition showed highly flourished silver dendrites. Here, we aimed to demonstrate the electrodeposited silver’s enhancement of the electrochemical signal. We chose an electrochemical deposition parameter by three cycles of CV scanning from 0 to −1.0 V at 25 mV s^−1^ and the total deposition time was about 240 s. Although it is difficult to distinguish the silver nanomaterials from the SEM images, the EDX spectra indicated that silver was successfully electrodeposited onto NSP–PC@GCE.

### 3.2. Electrochemical Sensing Performance

#### 3.2.1. Cyclic Voltammetry

**Figure 2:**
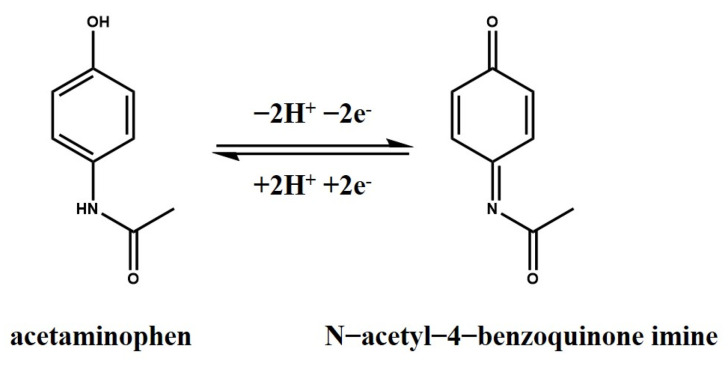
Redox reaction mechanism of APAP.

APAP’s redox reaction mechanism is shown in Figure 2.

**Figure 3:**
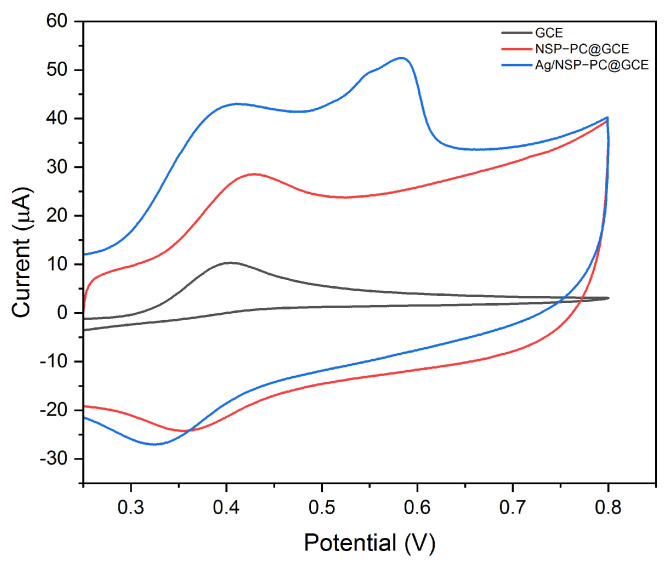
The CV curves of bare GCE (I_pa_ 10.8 μA), NSP–PC@GCE (I_pa_ 11.7 μA), and Ag/NSP–PC@GCE (I_pa_ 31.1 μA) in 500 μM APAP, 0.01 M PBS buffer pH 7.4, 50 mV s^−1^.

**Figure 4:**
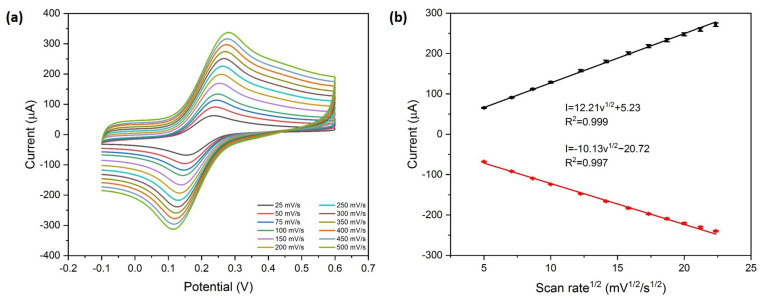
(**a**) CV curves of Ag/NSP–PC@GCE varied scan rates (25, 50, 75, 100, 150, 200, 250, 300, 350, 400, 450, and 500 mV s^−1^) in 5 mM [Fe(CN)_6_]^3−/4−^, 0.1 M KCl. (**b**) The linear fit of I_pa_ and I_pc_ versus the square root of the scan rate.

Three electrodes of bare GCE, NSP–PC@GCE, and Ag/NSP–PC@GCE were used for sensing 500 μM of APAP by CV scan; the curves are shown in Figure 3. All the electrodes displayed an oxidation peak near 0.4 V. For bare GCE, NSP–PC@GCE, and Ag/NSP–PC@GCE, the redox potentials and oxidation peak currents were, respectively, 0.404 V, I_pa_ 10.8 μA, 0.430 V, I_pa_ 11.7 μA, and 0.410 V, I_pa_ 31.1 μA. The potential position changed a little bit, while the current I_pa_ increased by about three times by the Ag/NSP–PC@GCE. There was another oxidation peak at about 0.585 V of the Ag/NSP–PC@GCE, which could be due to the partial oxidation of the electrodeposited Ag with dissolved oxygen or the chloride ion in the PBS buffer (Phosphate Buffered Saline) [25]. The potential position could be related to the electrodeposited Ag structure, e.g., particle size and structure, sphere, cubic, rod, or dendrite [26]. This peak remained inconspicuous after the first scan, as shown in Figure 4a as a very weak hump from 0.35 V to 0.55 V. The oxidized Ag (AgO_2_ or AgCl) remained on the surface of the non-oxidized Ag and functioned as a protective cover. The procedure of electrodeposition of silver could enhance the electrochemical signal due to silver’s high electrical conductivity. However, compared to other noble metals like gold or platinum, silver is much less stable and easily oxidized.

The electrochemical catalytic activity of the Ag/NSP–PC@GCE was examined by [Fe(CN)_6_]^3−/4−^ solution. By altering the scan rate, a series of CV curves in [Fe(CN)_6_]^3−/4−^ solution was obtained, as shown in Figure 4a. As the scan rate increased, the distance between redox peaks increased, and the redox current also increased. The Randles–Sevcik equation was used to estimate the surface area [27].

I_p_ = 2.69 × 10^5^ A D^1/2^ n^3/2^ v^1/2^ C (1)

where A is the electrochemical active surface area (cm^2^), D is the diffusion coefficient of the molecule in the bulk solution (6.67 × 10^−6^ cm^2^ s^−1^ for potassium ferricyanide), n is the number of electrons that participated in the reaction, v is the scan rate (V s^−1^), and C is the concentration of the bulk solution (M). The electrochemically active surface area of Ag/NSP–PC@GCE was calculated to be 0.113 cm^2^. For the bare GCE used in the experiments, the glassy carbon surface area is 0.07 cm^2^ (diameter 3 mm), and the Ag/NSP–PC@GCE has a 61.4% increase in surface area compared to bare GCE.

In Figure 4b, the current versus the square root of the scan rate is plotted. Both the I_pa_ and I_pc_ are linearly related to the square root of the scan rate, which indicates that the redox reaction of [Fe(CN)_6_]^3−/4−^ is a diffusion-controlled process. The linear fit equations from the graph are listed below.

I_pa_ = 12.21 × v^1/2^ + 5.23, R^2^ = 0.999 (2)

I_pc_ = 10.13 × v^1/2^−20.72, R^2^ = 0.997 (3)

#### 3.2.2. Electrochemical Impedance Spectroscopy

**Figure 5:**
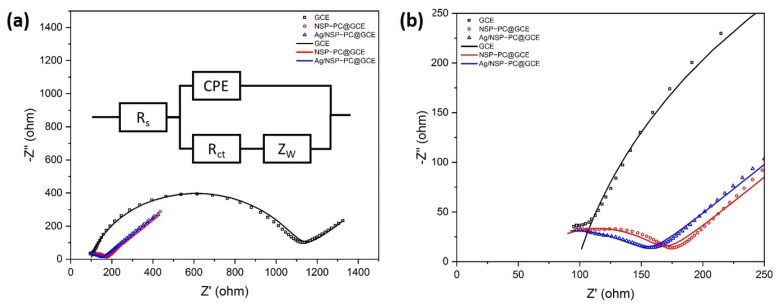
(**a**) EIS spectra of bare GCE, NSP–PC@GCE, and Ag/NSP–PC@GCE in 5 mM [Fe(CN)_6_]^3−/4−^, 0.1 M KCl, frequency range of 100 KHz to 1 Hz; (**b**) zoomed-in view of (**a**) in the range from 0 ohm to 250 ohm. The points are measured EIS data, and the curves are simulated curves.

The EIS measurement was carried out for the bare GCE, NSP–PC@GCE, and Ag/NSP–PC@GCE as shown in Figure 5a. The points were the measured EIS data, and the curves were the simulated data. Figure 5b is the zoomed-in view to show the data from 0 ohm to 250 ohm more clearly. The Randles equivalent circuit was used to simulate the Nyquist plot, and the charge transfer resistance (R_ct_) of bare GCE, NSP–PC@GCE, and Ag/NSP–PC@GCE were simulated to be, respectively, 1006 ohm, 107.3 ohm, and 124.3 ohm. In the Randles circuit, R_s_ represents the solution resistance, R_ct_ represents the charge transfer resistance, CPE represents the constant phase element, and Z_W_ represents the Warburg impedance. The good fit of the simulated curve confirmed that a suitable model was selected. The NSP–PC@GCE and Ag/NSP–PC@GCE both have a smaller R_ct_ compared to bare GCE. Additionally, after the electrodeposition of Ag, the R_ct_ increased a little. After the silver electrodeposition, some active sites might be deposited with silver, therefore causing an increase in the resistance. The EIS results suggested that porous carbon and silver electrodeposition could improve electron transfer speed and overall interfacial conductivity.

#### 3.2.3. APAP Concentration Dependence

**Figure 6:**
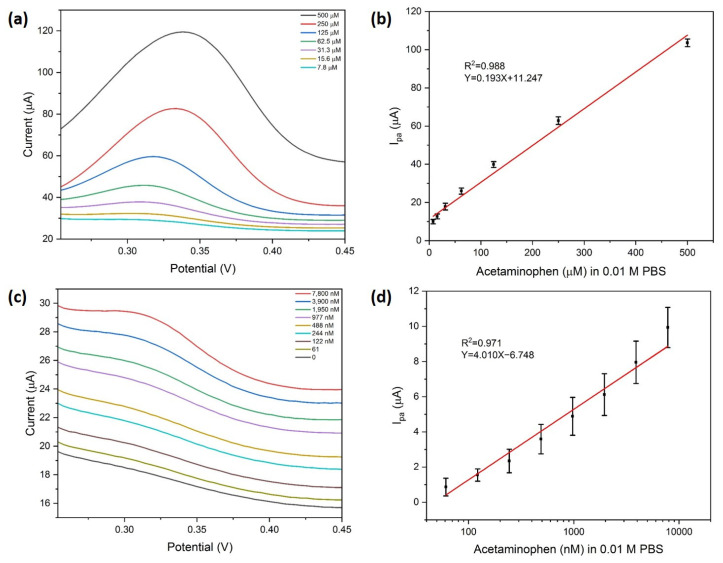
(**a**) DPV curves of Ag/NSP–PC@GCE sensing APAP in the concentration range of 7.8 μM to 500 μM, (**b**) linear fit curve of (**a**), (**c**) DPV curves of Ag/NSP–PC@GCE sensing APAP in the concentration range of 61 nM to 7.8 μM, (**d**) linear fit curve of (**c**), in 0.01 M PBS buffer pH 7.4, increment 0.004 V, amplitude 0.05 V, pulse width 0.05 s, sample width 0.0167 s, and pulse period 0.5 s.

The determination of APAP was performed using the DPV scan by Ag/NSP–PC@GCE. The results are shown in Figure 6, and two concentration ranges were achieved. The first concentration range is from 7.8 μM to 500 μM, from which a linear fit of I_pa_ versus APAP concentration was obtained. The fitting equation is shown below.

I_current_ = 0.193 × C_APAP_ + 11.247, R^2^ = 0.988 (4)

The second linear range is from 61 nM to 7.8 μM, from which the linear fit of DPV current versus the logarithm of APAP concentration was obtained. The fitting equation is shown below.

I_current_ = 4.010 × log_10_(C_APAP_) − 6.748, R^2^ = 0.971 (5)

The limit of detection of the second linear range was calculated based on the IUPAC 3σ principle to be 33 nM.

#### 3.2.4. Anti-Interference

**Figure 7:**
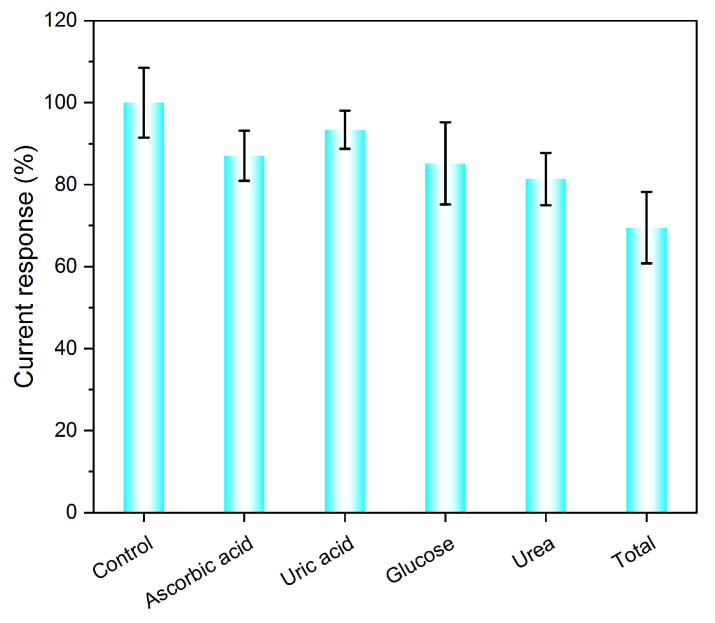
Current response (%) of Ag/NSP–PC@GCE sensing APAP in the presence of ascorbic acid, uric acid, glucose, urea, and a mixture of all interferences in 0.01 M PBS buffer pH 7.4, increment 0.004 V, amplitude 0.05 V, pulse width 0.05 s, sample width 0.0167 s, and pulse period 0.5 s.

The constructed Ag/NSP–PC@GCE was tested for anti-interference in multiple analyte solutions and a mixture of all analyte solutions. From Figure 7, the single interference has a small influence on APAP (125 μM). Under a single interference of ascorbic acid (125 μM), uric acid (125 μM), glucose (125 μM), and urea (125 μM), the current response (%) remained above 80% compared to the APAP control sample. The mixture of all the mentioned interference analytes (all interferences and APAP were 125 μM) resulted in about a 70% current response compared to the control sample. From the results, although the interference analytes have redox peaks at different potential positions, during the DPV scan, the interference analytes might have occupied some active spots and did not diffuse away from the sensing interface in time. The concentration of the interference was equal molar to the APAP. Therefore, a partial decrease in the current response was observed. In the mixture of all interference analytes, a total of four equal molar concentration interferences were present in the solution, which resulted in a further decrease in the current response of about 30%. However, under equal molar concentration interference, the anti-inference performance of Ag/NSP–PC@GCE was acceptable.

#### 3.2.5. Stability and Repeatability, Real Sample Measurement, and Comparison with Other Works

**Figure 8:**
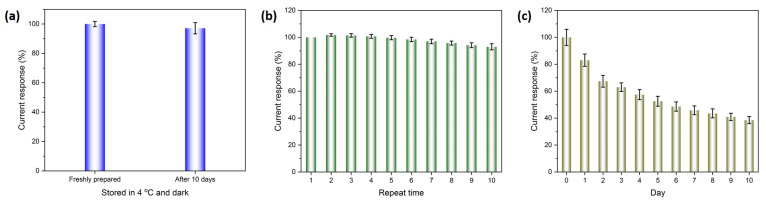
(**a**) Stability of the Ag/NSP–PC@GCE after 10 days of storage at 4 °C; (**b**) Repeated measurements of the Ag/NSP–PC@GCE; (**c**) Every day measurements of the Ag/NSP–PC@GCE; DPV measured in 0.01 M PBS buffer pH 7.4, increment 0.004 V, amplitude 0.05 V, pulse width 0.05 s, sample width 0.0167 s, pulse period 0.5 s.

The Ag/NSP–PC@GCE were prepared and stored in the fridge in darkness. After 10 days, the Ag/NSP–PC@GCE was taken out, and the measured results were compared to the freshly prepared Ag/NSP–PC@GCE. As shown in Figure 8a, the signal decreased to about 97.1 ± 3.8%, which demonstrated good stability of the Ag/NSP–PC@GCE. Next, we measured the Ag/NSP–PC@GCE continuously 10 times, as shown in Figure 8b; the 10th signal decreased to about 93%, which demonstrated good repeatability. Measurements taken every day were also carried out, as shown in Figure 8c. The 10th day measurement signal decreased to about 40%, which shows that the Ag/NSP–PC@GCE could not stand for days after measurement. Because the porous carbon was dropped cast onto the GCE surface with electrodeposited silver, no further surface fixing or protective steps like Nafion solutions were applied. For 10 days of measurement, the GCE was stored at room temperature in darkness. The repeating wet-dry condition may cause physicochemical changes to the sensing interface, e.g., some porous carbon might fall off, further oxidation of the electrodeposited silver, and loss of electroactive spots.

The human urine sample was provided by healthy male volunteers who did not take medicine containing APAP for at least a month in the lab. The urine was cooled to room temperature and used directly for measurement. Four samples were tested: 100 times diluted, 50 times diluted, 10 times diluted, and undiluted samples. The results are summarized in Table 1. In research by the authors of [4] APAP concentration in urine, the highest quartile (>73.5 ng/mL, 486 μM) and the lowest quartile (<5.44 ng/mL, 36 μM) of male urinary paracetamol were studied. For the urine samples, we chose the spiked APAP concentration to be around 200 μM to 400 μM. As seen from the summary results in Table 1, the undiluted urine sample has a lower recovery rate compared to the diluted samples. Additionally, two peaks were present in the DPV curves of the undiluted sample. The other peak was speculated to come from uric acid after comparing the DPV curves with reference [19] and the DPV curves from the anti-interference curves. As the clinical uric concentration in urine is in the range of 1 mM to 4.4 mM [28], the content of uric acid in the undiluted urine sample would be many times more concentrated compared to the spiked APAP. While diluted samples (100, 50, and 10 times dilution) did not appear in the two peaks. Several other factors might also affect the sensor’s performance, like urine pH and other interference compositions such as protein and ions present in the urine. These interferences might be adsorbed onto the Ag/NSP–PC@GCE surface. Therefore, for the undiluted urine sample, a lowered recovery rate of 87 ± 13% was obtained.

If the urine sample is 100 times diluted, the acetaminophen concentration in a real urine sample would be 20 mM, which would be a very high concentration. Based on reference [4], in male urine, the APAP highest quartile is >73.47 ng/mL (0.486 mM). Based on the research of the mathematical model for APAP metabolism study by Nijhout [29], if the dose of APAP is 26.5 mmole (4 g), the APAP in urine would be about 2 mM over a 24 h accumulation, the APAP metabolites of APAP-S in urine would be 1.8 mM, APAP-G in urine would be 12 mM, and some APAP and its metabolites would be distributed in plasma and tissues. The total amount of APAP, APAP-S, and APAP-G in urine would be about 16 mM, which means that about 60% of the dose of APAP (26.5 mmol) would be excreted by urine after 24 h. According to the US Food and Drug Administration’s suggestion [30], the current maximum recommended adult dose of acetaminophen is 4 g per day. Therefore, if the APAP in urine is 20 mM, it would not be normal for pain and fever treatment. Based on Nijhout’s study [29], if the dose was less than 20 g, the application of an antidote of N-acetylcysteine (NAC) within 8 h of an overdose of APAP is usually successful in preventing liver failure. However, the 20 g dose of APAP would lead to hepatocyte depletion to about 30%, which is considered to be at the critical edge of liver failure. Assuming that the intake of 40 g of APAP would result in 20 mM APAP in urine, based on Nijhout’s study [29], the liver would be seriously damaged, and the patient is probably unable to be rescued even using the antidote NAC.

A survey of recent references to APAP sensing is summarized in Table 2. This study covered a wider concentration range from 61 nM to 500 μM, with a low detection limit of 33 nM. Some of the references have a lower limit of detection but not a wider sensing range. This study complements the APAP sensing range with a good limit of detection.

## 4. Conclusions

In summary, through the facile doping of nitrogen, sulfur, and phosphorus elements for electrical conductivity enhancement, nanostructured Elaeagnus Angustifolia gum derived porous biomass carbon material was prepared. This porous carbon material was dropped onto GCE with in situ electrodeposition of silver for the enhancement of electrochemical sensing towards APAP with good sensitivity and selectivity. Two linear ranges from 61 nM to 500 μM were achieved with a limit of detection of 33 nM. The developed GCE sensor has good anti-interference against several chemicals, including ascorbic acid, uric acid, glucose, and urea. Good stability and reproducibility under fridge storage for 10 days were confirmed. This study demonstrated the promising application of the nanostructured Elaeagnus Angustifolia gum-derived porous biomass carbon material with silver electrodeposition as a potential electrode material for electrochemical sensing.

## Figures and Tables

**Table 1:** Detection result of spiked APAP in urine samples (n ≥ 3).

Sample	Species	Added (μM)	Found (μM)	Recovery (%)
Urine ^1^	APAP	200	211 ± 27	106 ± 14
Urine ^2^	APAP	200	201 ± 35	101 ± 18
Urine ^3^	APAP	400	392 ± 40	98 ± 10
Urine ^4^	APAP	300	261 ± 39	87 ± 13

^1^ diluted by 100 times in 0.01 M PBS buffer, pH 7.4. ^2^ diluted by 50 times in 0.01 M PBS buffer, pH 7.4. ^3^ diluted by 10 times in 0.01 M PBS buffer, pH 7.4. ^4^ undiluted.

**Table 2:** Comparison of other electrode materials for APAP determination.

Electrode Material	Sensing Range (μM)	Limit of Detection (μM)	Reference
ZnCl_2_-KOH activated kelp carbon	0.01–20	0.004	[2]
hollow nanoporous carbon polyhedrons embedded with Co/Co_3_O_4_ nanoparticles	0.025−50	0.0083	[18]
N, P-doped hollow mesoporous carbon nanospheres	5–1200	0.02	[19]
Bismuth oxide nanostructures	0.5–1250	0.03	[20]
Ag/Ag-oxide-graphene	9.9–64.9	0.022	[31]
Ni/NiO/Ni–B/graphene heterostructure	10–2500	14	[32]
Ni_2_P nanosheets	0.5–4500	0.107	[33]
Barium titanate nanocubes	10–100	0.23	[34]
Ferrocene-reduced graphene oxide-polyoxometalates nanocomposites	1–1000	0.013	[35]
Hollow Fe_3_O_4_-rGO nanocomposites	0.5–500	0.11	[36]
Ag/NSP–PC	0.061–500	0.033	This work

## Data Availability

The data is available on reasonable request from the corresponding author.

## References

[1] Chen L., Yang Y., Ueno H., Esch M.B. (2020). Body-in-a-Cube: A microphysiological system for multi-tissue co-culture with near-physiological amounts of blood surrogate. Microphysiol. Syst..

[2] Kim D., Kim J.M., Jeon Y., Lee J., Oh J., Hooch Antink W., Kim D., Piao Y. (2018). Novel two-step activation of biomass-derived carbon for highly sensitive electrochemical determination of acetaminophen. Sens. Actuators B Chem..

[3] Alam A.U., Qin Y., Catalano M., Wang L., Kim M.J., Howlader M.M.R., Hu N.-X., Deen M.J. (2018). Tailoring MWCNTs and β-Cyclodextrin for Sensitive Detection of Acetaminophen and Estrogen. ACS Appl. Mater. Interfaces.

[4] Smarr M.M., Grantz K.L., Sundaram R., Maisog J.M., Honda M., Kannan K., Buck Louis G.M. (2016). Urinary paracetamol and time-to-pregnancy. Hum. Reprod..

[5] Labib M., Sargent E.H., Kelley S.O. (2016). Electrochemical Methods for the Analysis of Clinically Relevant Biomolecules. Chem. Rev..

[6] Huang T.-Y., Kung C.-W., Wei H.-Y., Boopathi K.M., Chu C.-W., Ho K.-C. (2014). A high performance electrochemical sensor for acetaminophen based on a rGO–PEDOT nanotube composite modified electrode. J. Mater. Chem. A.

[7] Mehmandoust M., Li G., Erk N. (2022). Biomass-Derived Carbon Materials as an Emerging Platform for Advanced Electrochemical Sensors: Recent Advances and Future Perspectives. Ind. Eng. Chem. Res..

[8] Wang Y., Zhang M., Shen X., Wang H., Wang H., Xia K., Yin Z., Zhang Y. (2021). Biomass-Derived Carbon Materials: Controllable Preparation and Versatile Applications. Small.

[9] Xu C., Liu J., Bi Y., Ma C., Bai J., Hu Z., Zhou M. (2020). Biomass derived worm-like nitrogen-doped-carbon framework for trace determination of toxic heavy metal lead (II). Anal. Chim. Acta.

[10] Wang L., Zhang Q., Chen S., Xu F., Chen S., Jia J., Tan H., Hou H., Song Y. (2014). Electrochemical Sensing and Biosensing Platform Based on Biomass-Derived Macroporous Carbon Materials. Anal. Chem..

[11] Veerakumar P., Koventhan C., Chen S.-M. (2023). Copper-palladium alloy nanoparticles immobilized over porous carbon for voltammetric determination of dimetridazole. J. Alloys Compd..

[12] Lu Z., Li S., Li Y., Li L., Ma H., Wei K., Shi C., Sun M., Duan R., Wang X. (2023). DFT-assisted design inspired by loofah-derived biomass carbon decorated CoFe-CoFe_2_O_4_ conjugated molecular imprinting strategy for hazardous thiamphenicol analysis in spiked food. Sens. Actuators B Chem..

[13] Malode S.J., Shanbhag M.M., Kumari R., Dkhar D.S., Chandra P., Shetti N.P. (2023). Biomass-derived carbon nanomaterials for sensor applications. J. Pharm. Biomed. Anal..

[14] Abbas A., Amin H.M.A. (2022). Silver nanoparticles modified electrodes for electroanalysis: An updated review and a perspective. Microchem. J..

[15] Song W., Li H., Liang H., Qiang W., Xu D. (2014). Disposable Electrochemical Aptasensor Array by Using In Situ DNA Hybridization Inducing Silver Nanoparticles Aggregate for Signal Amplification. Anal. Chem..

[16] Zhu Y., Chandra P., Shim Y.-B. (2013). Ultrasensitive and Selective Electrochemical Diagnosis of Breast Cancer Based on a Hydrazine–Au Nanoparticle–Aptamer Bioconjugate. Anal. Chem..

[17] Sun D., Yang D., Wei P., Liu B., Chen Z., Zhang L., Lu J. (2020). One-Step Electrodeposition of Silver Nanostructures on 2D/3D Metal–Organic Framework ZIF-67: Comparison and Application in Electrochemical Detection of Hydrogen Peroxide. ACS Appl. Mater. Interfaces.

[18] Wang K., Wu C., Wang F., Jing N., Jiang G. (2019). Co/Co_3_O_4_ Nanoparticles Coupled with Hollow Nanoporous Carbon Polyhedrons for the Enhanced Electrochemical Sensing of Acetaminophen. ACS Sustain. Chem. Eng..

[19] Mahanthappa M., Duraisamy V., Arumugam P., Senthil Kumar S.M. (2022). Simultaneous Determination of Ascorbic Acid, Dopamine, Uric Acid, and Acetaminophen on N, P-Doped Hollow Mesoporous Carbon Nanospheres. ACS Appl. Nano Mater..

[20] Mahmoud B.G., Khairy M., Rashwan F.A., Banks C.E. (2017). Simultaneous Voltammetric Determination of Acetaminophen and Isoniazid (Hepatotoxicity-Related Drugs) Utilizing Bismuth Oxide Nanorod Modified Screen-Printed Electrochemical Sensing Platforms. Anal. Chem..

[21] Chen L., Mamat X., Aisa H.A. (2023). Determination of aflatoxin B_1_ by biomass derived porous carbon modified electrode with molecularly imprinted polymer. Electroanalysis.

[22] Yalikun N., Mamat X., Li Y., Hu X., Wang P., Hu G. (2019). N, S, P-Triple Doped Porous Carbon as an Improved Electrochemical Sensor for Metronidazole Determination. J. Electrochem. Soc..

[23] Wang G., Yayalikun N., Mamat X., Li Y., Hu X., Wang P., Xin X., Hu G. (2020). Highly Sensitive Electrochemical Sensor for the Detection of Chloramphenicol Based on Biomass Derived Porous Carbon. Sci. Adv. Mater..

[24] Mandke M.V., Han S.-H., Pathan H.M. (2012). Growth of silver dendritic nanostructuresvia electrochemical route. CrystEngComm.

[25] Qin X., Liu L., Xu A., Wang L., Tan Y., Chen C., Xie Q. (2016). Ultrasensitive Immunoassay of Proteins Based on Gold Label/Silver Staining, Galvanic Replacement Reaction Enlargement, and In Situ Microliter-Droplet Anodic Stripping Voltammetry. J. Phys. Chem. C.

[26] Ivanova O.S., Zamborini F.P. (2010). Size-Dependent Electrochemical Oxidation of Silver Nanoparticles. J. Am. Chem. Soc..

[27] Bard A.J., Faulkner L.R. (2011). Electrochemical Methods: Fundamentals and Applications.

[28] Kulyk B., Pereira S.O., Fernandes A.J.S., Fortunato E., Costa F.M., Santos N.F. (2022). Laser-induced graphene from paper for non-enzymatic uric acid electrochemical sensing in urine. Carbon.

[29] Ben-Shachar R., Chen Y., Luo S., Hartman C., Reed M., Nijhout H.F. (2012). The biochemistry of acetaminophen hepatotoxicity and rescue: A mathematical model. Theor. Biol. Med. Model..

[30] US Food & Drug Administration. https://www.fda.gov/consumers/consumer-updates/dont-double-acetaminophen.

[31] Bhat S.A., Rather M.A., Pandit S.A., Ingole P.P., Bhat M.A. (2016). Sensitive electrochemical sensing of acetaminophen and hydroquinone over single-pot synthesized stabilizer free Ag/Ag-oxide-graphene nanocomposites. J. Electrochem. Soc..

[32] Ipekci H.H., Ozcan M., Turkyilmaz B.G., Uzunoglu A. (2021). Ni/NiO/Ni–B/graphene heterostructure-modified electrodes and their electrochemical activities towards acetaminophen. Anal. Methods.

[33] Wei M., Lu W., Liu G., Jiang Y., Liu X., Bai L., Cao X., Jia J., Wu H. (2021). Ni_2_P Nanosheets: A High Catalytic Activity Platform for Electrochemical Detection of Acetaminophen. Chin. J. Chem..

[34] Ali M., Sharma S., Singh R., Sharma K., Majhi S., Guin D., Tripathi C.S.P. (2022). Barium Titanate Nanocubes as a Dual Electrochemical Sensor for Detection of Dopamine and Acetaminophen. J. Electrochem. Soc..

[35] Han H., Liu C., Sha J., Wang Y., Dong C., Li M., Jiao T. (2021). Ferrocene-reduced graphene oxide-polyoxometalates based ternary nanocomposites as electrochemical detection for acetaminophen. Talanta.

[36] Shen L., Dong J., Wen B., Wen X., Li J. (2023). Facile Synthesis of Hollow Fe_3_O_4_-rGO Nanocomposites for the Electrochemical Detection of Acetaminophen. Nanomaterials.

